# Case Report: Changes in Regional Cerebral Blood Flow in Chronic Akathisia of a Depressed Patient Before and After Electroconvulsive Therapy Treatment

**DOI:** 10.3389/fpsyt.2021.728265

**Published:** 2021-09-10

**Authors:** Akihito Suzuki, Ryota Kobayashi, Toshinori Shirata, Hitomi Komoriya, Masafumi Kanoto, Koichi Otani

**Affiliations:** ^1^Department of Psychiatry, Yamagata University School of Medicine, Yamagata, Japan; ^2^Department of Diagnostic Radiology, Yamagata University Faculty of Medicine, Yamagata, Japan

**Keywords:** depressive disorder, chronic akathisia, electroconvulsive therapy (ECT), regional cerebral blood flow, SPECT

## Abstract

Akathisia, which characterized by subjective restlessness and objective hyperactivity, is induced mostly by antipsychotics and antidepressants. Chronic akathisia is defined as persistence of symptoms for more than 3 months. The pathophysiology of chronic akathisia remains unclear. This report describes a depressed patient, a 66-year-old woman with a diagnosis of major depressive disorder, with chronic akathisia. Her regional cerebral blood flow (rCBF) was measured using single photon emission computed tomography (SPECT) before and after the treatment with electroconvulsive therapy (ECT). She had experienced akathisia-like symptoms three times prior because of risperidone, escitalopram, and clomipramine administration, accompanied by major depression. After levomepromazine was added to quetiapine to treat insomnia, she developed akathisia symptoms such as a sense of restlessness and inability to sit in one place for a few minutes. These antipsychotics were withdrawn. Propranolol was administered, leading to no apparent improvement for 8 months. After she was diagnosed as having major depressive disorder and chronic akathisia, she received 10 sessions of bilateral ECT. Her depressive symptoms improved greatly. Akathisia disappeared completely after ECT. SPECT revealed that rCBF was decreased in the middle frontal gyrus and parietal lobe, that it was increased in the thalamus, fusiform gyrus, and cerebellum before ECT, and that these abnormalities in rCBF were approaching normal levels after ECT. Findings presented in this report suggest ECT as a beneficial treatment for chronic akathisia. Altered rCBF in the middle frontal gyrus, parietal lobe, thalamus, fusiform gyrus, and cerebellum, and especially decreased rCBF in the parietal lobe, may be related to the pathophysiology of chronic akathisia.

## Introduction

Akathisia is characterized by subjective symptoms, that is, a feeling of restlessness and inner tension, and the objective symptoms, that is, purposeless leg movements and inability to sit for several minutes. Akathisia is induced mostly by antipsychotics and antidepressants (1–3). According to Sachdev (2), akathisia is classified as acute, tardive, withdrawal, or chronic. Acute akathisia is defined as rapid onset within hours or days after exposure to causative drugs. Hypoactivity of dopamine in the nigrostriatal and mesolimbic pathways, hyperactivities in noradrenaline and serotonin pathways, and reduced glucose uptake in the thalamus and cerebellum are inferred as factors of acute akathisia development (4, 5). Chronic akathisia is defined as persistence of akathisia symptoms for more than 3 months, even after treatment discontinuation or dosage reduction (1, 2). For the treatment of chronic akathisia, several case reports have described the beneficial use of mianserin (6), clonidine (7), propranolol (8), and electroconvulsive therapy (ECT) (9). However, no report describes examination of the pathophysiology of chronic akathisia including brain imaging. This report describes a depressed patient with chronic akathisia whose regional cerebral blood flow (rCBF) was measured using single photon emission computed tomography (SPECT) before and after treatment with ECT.

## Case Report

The case was that of a 66-year-old Japanese woman. She gave written informed consent for the reporting her clinical course. The Ethics Committee of Yamagata University School of Medicine approved this report. She had no past history of neurological disease. She had surgery for breast cancer at 59 years old without further treatment such as radiation or chemotherapy. Then she was treated using antihistamine drugs for psoriasis vulgaris. Her daughter had intellectual disability, but there was no other family history.

**Figure 1:**
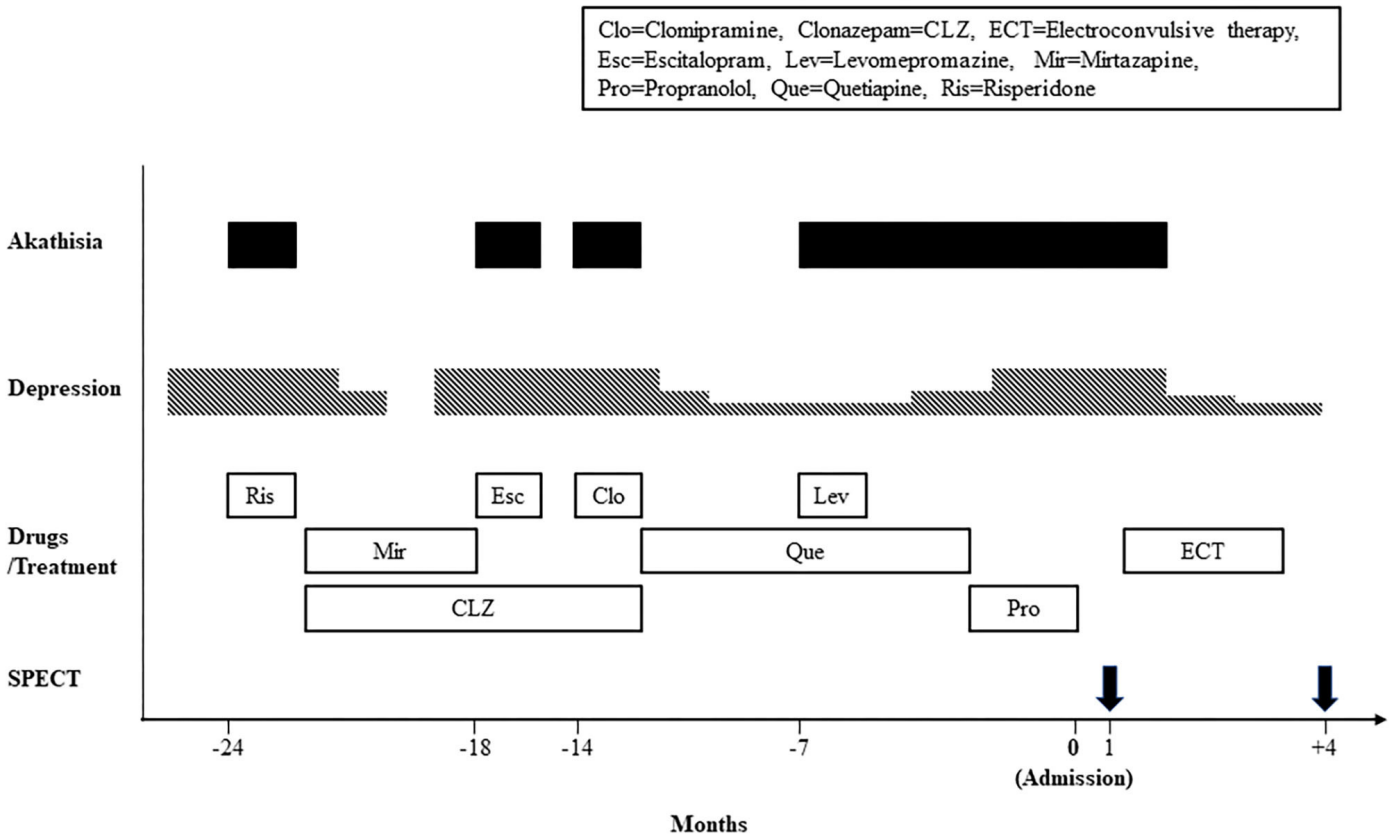
Timeline of the clinical course.

Figure 1 shows the timeline of the clinical course. In 24 months before admission to our hospital, she developed anxiety, depressed mood, and insomnia, and began treatment with risperidone 0.5 mg/day at a mental clinic. Two days after treatment initiation, she displayed a sense of restlessness and an inability to sit calmly. After she was referred to a psychiatric hospital, she was diagnosed as having major depression and akathisia. She was treated with mirtazapine 30 mg/day and clonazepam 0.5 mg/day, leading to complete remission. In 18 and 14 months before admission, her depression relapsed twice, for which she was respectively administered escitalopram 10 mg/day and clomipramine 12.5 mg/day. Immediately after taking these drugs, she displayed akathisia. These drugs were withdrawn. Later she was treated with quetiapine 25 mg/day with mild depressive symptoms because she refused any treatment by antidepressants. In 7 months before admission to our hospital, her akathisia symptoms were emerged again, immediately after levomepromazine 25 mg/day was added to treat insomnia. After antipsychotic drugs were withdrawn for 2 months, propranolol was administered at a dosage of up to 60 mg/day for akathisia and hypertension, with no apparent improvement. She was referred to our hospital to undergo ECT.

On admission, she felt a sense of restlessness, inner tension, and a constant wish to move the legs. Objectively, she displayed an inability to sit in one place for a few minutes, rocking from foot to foot, stamping, and pacing. It is particularly interesting that she and her husband reported that her feeling of restlessness ceased when she was in a moving car driven by her husband. Therefore, they spent 3–6 h per day in the car. These akathisia symptoms led to her suicidal thoughts and her inability to concentrate on any activity such as reading books or watching TV. She also had depressive symptoms such as depressed mood, insomnia, reduced appetite, and pessimistic thoughts. She did not have extrapyramidal symptoms such as muscle rigidity, resting tremor, akinesia, and gait disturbance. She scored 28/30 on the Mini-Mental State Examination. Her laboratory blood tests and brain magnetic resonance imaging were unremarkable. To exclude hyperkinesia because of Parkinson disease, the dopamine transporter availability was measured using ^123^I-N-fluoropropyl-2b-carbomethoxy-3b- (4-iodophenyl) nortropane SPECT, with no abnormality. She was diagnosed as having major depressive disorder and chronic akathisia respectively according to the DSM-5 (1) and criteria for akathisia (2). She scored 45/60 on the Montgomery-Asberg Depression Rating Scale (MADRS) (10) and 4/5 (marked akathisia) on the Barnes Akathisia Rating Scale (BARS) (3). She received 10 sessions of bilateral ECT, using a Tymatron system IV machine (Somatics, LLC, Venice, FL, USA). The ECT treatments were performed twice a week according to the procedure using a 0.5-mS pulse width with frequency of 30 Hz, stimulus duration of 5.6 s, current of 900 mA, and with a maximal charge output of 151.3 mC. Anesthesia was induced with propofol and muscle relaxation was performed using rocuronium bromide. After five sessions of ECT, her depressive symptoms were improved gradually to scores of 22/60 of the MADRS. Her akathisia symptoms disappeared completely to 0/5 of the BARS. After 10 ECT sessions, she scored 9/60 on the MADRS and 0/5 on the BARS. In 4 months after admission, she was discharged. In 5 months after admission, she was given clonazepam 1 mg/day and brotizolam 0.25 mg/day. She still remitted, with scores of 3/60 of the MADRS and 0/5 of the BARS.

**Figure 2:**
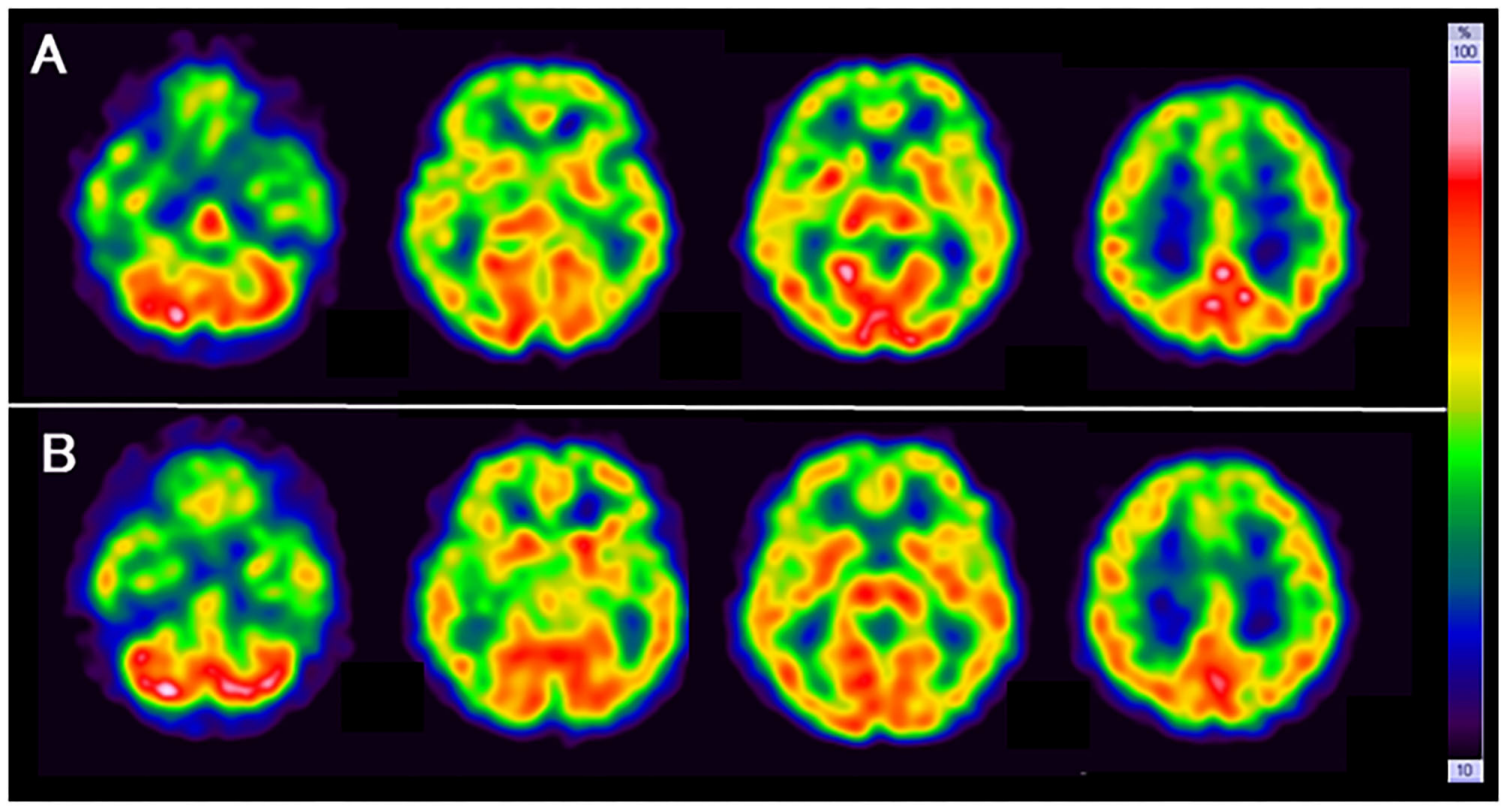
SPECT images before ECT (**A**, upper) and after ECT (**B**, lower).

**Figure 3:**
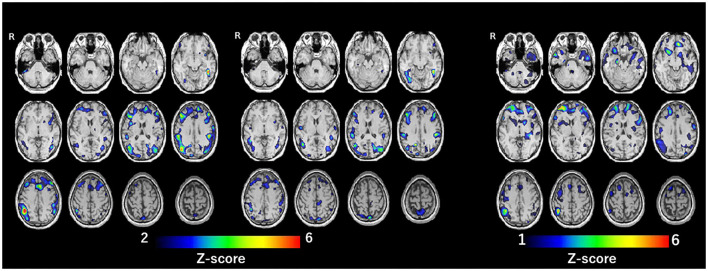
Reduction of rCBF: before ECT (**A**, left), after ECT (**B**, center), and difference of rCBF before and after ECT (regions where rCBF reduction improved) (**C**, right).

**Figure 4:**
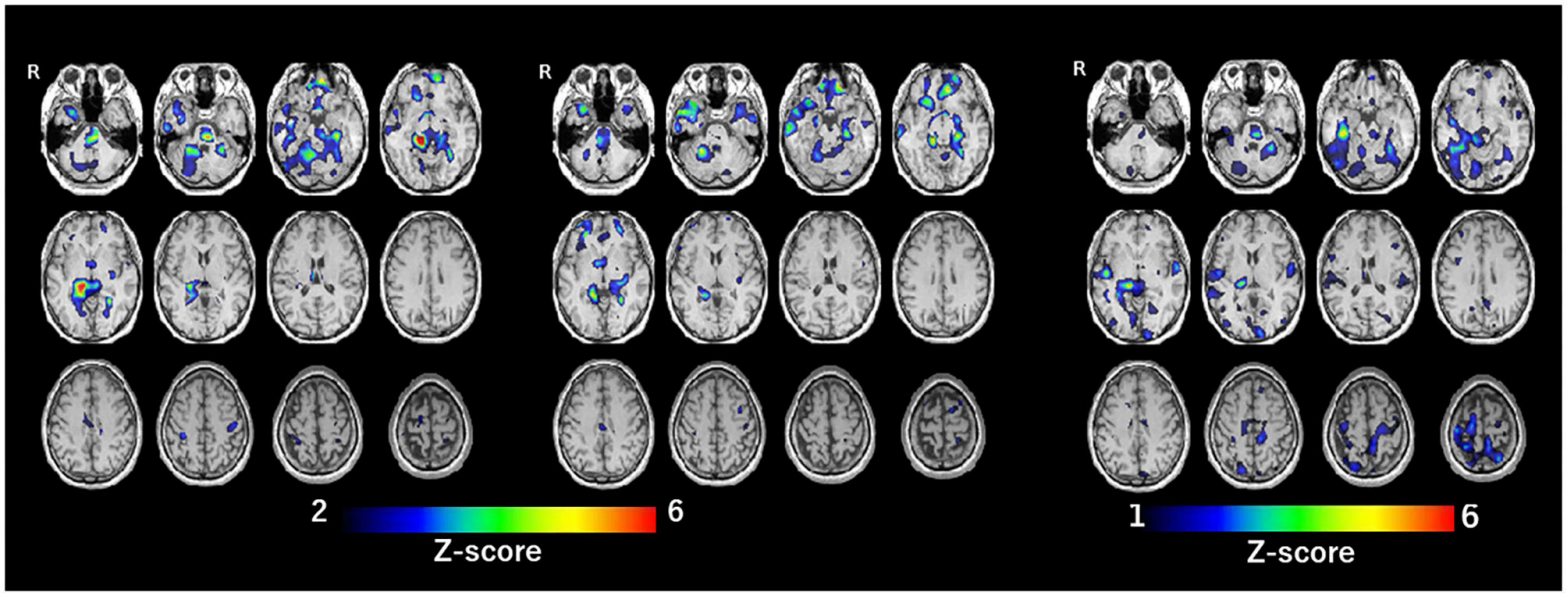
Increase of rCBF before ECT (**A**, left), after ECT (**B**, center), and difference of rCBF before and after ECT (regions where rCBF increase improved) (**C**, right).

SPECT with ^99m^Tc-ethylcysteinate dimer was performed before the beginning of ECT (in 1 month after admission) (A) and after the end of ECT (B) (in 4 months after admission) (Figure 1). SPECT imaging data were analyzed using the easy Z-score imaging system (FUJIFILM Toyama Chemical. Tokyo, Japan) compared with 40 age-matched normal controls. In addition, we used the voxel-based stereotactic extraction estimation (FUJIFILM Toyama Chemical. Tokyo, Japan) to compute the extent of rCBF reduction or increase for each region of interest before and after ECT and displayed its differences. Figures 2A,B show SPECT images before ECT (A) and after ECT (B). Figures 3A,B show the reduction in rCBF before ECT (A) and after ECT (B). Figure 3C shows changes in rCBF reduction on SPECT before and after ECT (regions where rCBF reduction improved). The decrease in rCBF in the middle frontal gyrus and parietal lobe before ECT was ameliorated after ECT (Figures 3A–C). Figures 4A,B show the increase in rCBF before ECT (A) and after ECT (B). Figure 4C shows changes in rCBF increase on SPECT before and after ECT (regions where rCBF increase improved). The increase in rCBF in the thalamus, fusiform gyrus, and cerebellum before ECT approached normal levels after ECT (Figures 4A–C).

## Discussion

In the present case, it might be difficult to distinguish chronic akathisia from agitation of major depressive disorder or from anxiety disorders, because symptoms of major depression and anxiety disorders can be confused with the objective and subjective symptoms of akathisia, respectively. However, it is suggested that a definite diagnosis of akathisia, unlike these psychiatric symptoms, should be made only if at least one subjective symptom and one objective symptom (2, 3, 11), which were eminently present in this case. In addition, akathisia symptoms observed in this case were not always parallel to the severity of her depression. Therefore, it is likely that her akathisia differed from agitation of major depression or anxiety disorders.

The treatment of ECT led to almost complete remission of depression and chronic akathisia in the present case. This finding is in line with a case report demonstrating the effectiveness of ECT for tardive and chronic akathisia in a patient of treatment-resistant depression (9). Consequently, the findings suggest that ECT may be beneficial treatment for chronic akathisia accompanied with major depression.

Along with the clinical course of the present case, rCBF before ECT was decreased in the middle frontal gyrus and parietal lobe and was increased in the thalamus, fusiform gyrus, and cerebellum. Also, rCBF in these regions approached normal levels after ECT. This report is the first to show brain imaging data in a patient with chronic akathisia. Results of meta-analysis suggest that patients with medication-free major depression show altered rCBF in the frontal-limbic-thalamic-striatal neural circuit, notably decreased rCBF in the middle frontal gyrus and increased rCBF in the thalamus (12). Thus, it is possible that rCBF reduction in the middle frontal gyrus and rCBF increase in the thalamus of the present case before ECT might be ascribable to major depression, and that altered rCBF in these regions was improved after ECT, together with the amelioration of depression. A case report by Smith (13) described that chronic akathisia in a patient of bipolar disorder was abolished by passive motion during travel as a car passenger. Coincidentally, the present patient also reported the same phenomenon, that is, ameliorated akathisia in a moving car. In describing a report of Smith (13), Patel et al. (14) suggested that a perception of movement via visual and vestibular input improves the sensory discomfort of akathisia and thus, disturbances in sensorimotor integration may be involved in the pathophysiology of akathisia. Furthermore, the parietal lobe was shown to play a crucially important role in higher-order processes of sensory inputs, and multisensory and sensorimotor integration (15). Therefore, findings from the case described herein suggest that dysfunction of the parietal lobe, that is, decreased rCBF in the parietal lobe, may be related to the pathophysiology of chronic akathisia.

## Conclusion

The findings reported herein suggest that ECT is a beneficial treatment for chronic akathisia. Altered rCBF in the middle frontal gyrus, parietal lobe, thalamus, fusiform gyrus, and cerebellum, especially decreased rCBF in the parietal lobe, may be related to the pathophysiology of chronic akathisia.

## Data Availability Statement

The original contributions presented in the study are included in the article/supplementary material, further inquiries can be directed to the corresponding authors.

## Ethics Statement

Written informed consent was obtained from the individual(s) for the publication of any potentially identifiable images or data included in this article.

## Author Contributions

AS, TS, and HK were responsible for clinical care. AS drafted the manuscript. RK critically revised the manuscript and analyzed SPECT with 99mTc-ethylcysteinate dimer. KO supervised clinical care and critically revised the manuscript. All authors contributed to the article and approved the submitted version.

## Conflict of Interest

The authors declare that the research was conducted in the absence of any commercial or financial relationships that could be construed as a potential conflict of interest.

## Publisher's Note

All claims expressed in this article are solely those of the authors and do not necessarily represent those of their affiliated organizations, or those of the publisher, the editors and the reviewers. Any product that may be evaluated in this article, or claim that may be made by its manufacturer, is not guaranteed or endorsed by the publisher.
